# Abdominal Pannus Should Not Dictate Surgical Approach in Primary Total Hip Arthroplasty

**DOI:** 10.1016/j.artd.2025.101675

**Published:** 2025-03-26

**Authors:** Samantha Bialek, William Oetojo, Robert Burnham, Nicholas Brown

**Affiliations:** Loyola University Medical Center, Department of Orthopaedic Surgery & Rehabilitation, Maywood, IL, USA

**Keywords:** Total hip arthroplasty, Surgical approach, Abdominal pannus, Obesity, Delayed wound healing, Infection risk

## Abstract

**Background:**

Increased pannus size is a risk factor for complications with anterior approach total hip arthroplasty (THA). However, it is unclear if changing to a posterior approach mitigates this risk. The purpose of this study was to evaluate whether abdominal pannus size had a differential effect on complication rate comparing anterior vs posterior THA.

**Methods:**

One thousand consecutive primary THA patients—478 anterior and 522 posterior—were retrospectively reviewed for complications and their abdominal pannus was radiographically measured on an anteroposterior pelvis image and placed into 1 of 4 categories based on its vertical size (no pannus [G0], above symphysis [G1], below symphysis [G2], or below ischial tuberosities [G3]). Chi-squared tests for univariate and logistic regression models controlled for age, race, gender, body mass index, Charlson comorbidity index, and smoking.

**Results:**

Comparing wound complications at increasing pannus size, anterior vs posterior (G0 1.9% vs 3.9%, *P* = .21; G1 7.2% vs 6.7%, *P* = .08; G2 17.9% vs 11.6%, *P* = .27; G3 16.7% vs 15.5%, *P* = .84), similar results were found with reoperations (G0 0.9% vs 1.1%, *P* = .080; G1 1.4% vs 2%, *P* = .72; G2 3.0% vs 5.8%, *P* = .41; G3 1.7% vs 4.5%, *P* = .33). Additionally, logistic regression models demonstrated no statistically significant difference in the odds of wound complications or reoperations between the approaches at each pannus size.

**Conclusions:**

In patients with an abdominal pannus, there is no difference in the risk of delayed wound healing or reoperation within 90 postoperative days comparing anterior to posterior approach.

## Introduction

Some arthroplasty surgeons prefer to perform total hip arthroplasties (THAs) from a posterolateral approach when encountering patients with a high body mass index (BMI) and large abdominal pannus. In 2016, Purcell et al first documented significantly increased rates of postoperative infection after direct anterior approach hip arthroplasty in patients with a BMI > 35 [1]. Since then, multiple studies have documented the increased infection rates in obese patients with this approach [[1], [2], [3], [4], [5], [6]].

The main theory behind this phenomenon is that patients who have elevated BMIs and an abdominal pannus likely suffer from some degree of immune dysregulation; combined with the proximity of the incision to the pannus, could lead to elevated risks for postoperative infection [1]. Consequentially, other comorbidities that often coexist with obesity, such as diabetes, may also increase the risk for postoperative infection [4]. Saini et al has noted that the size of pannus, namely if the pannus extended past the upper pubic symphysis, correlated with increased surgery complexity as well as postoperative infection and fractures in anterior approach hip arthroplasty [3].

With this seemingly significant association among pannus, obesity, and postoperative complication, studies have also investigated prophylactic measures and treatments for these patients—specifically, bariatric surgery prior to THA. However, per Smith et al, bariatric surgery prior to arthroplasties did not confer a significant change in rates of wound infection or revision surgery [7]. Furthermore, recent studies have begun to study the validity of prior theories. Purcell et al discovered no differences in rates of deep infection in patients with varying BMIs after THA via direct anterior and posterior approach [8]. They also noted an increased rate of superficial wound infection in patients across all BMIs who received the direct anterior approach [8]. Di Martino et al conducted a study similarly demonstrating that obese patients did not show higher rates of infection after direct anterior approach compared to patients with lower BMI [9]. Obese patients in the study by Di Martino et al did note an increased rate of revision; however, these were due to noninfectious etiologies (2 aseptic loosening, 1 dislocation, and 1 postoperative leg length discrepancy) [9].

The goal of this study was to investigate if increasing abdominal pannus size has a differential effect on complication rate when comparing anterior vs posterior THA.

## Material and methods

**Figure 1:**
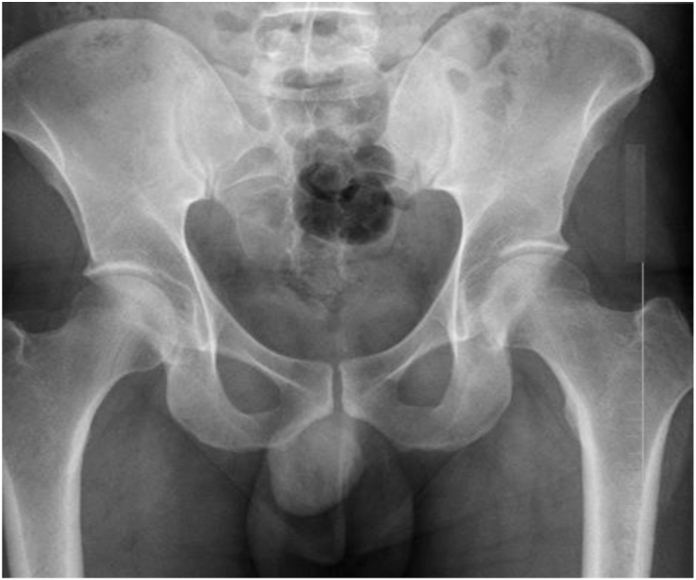
G0, no pannus.

**Figure 2:**
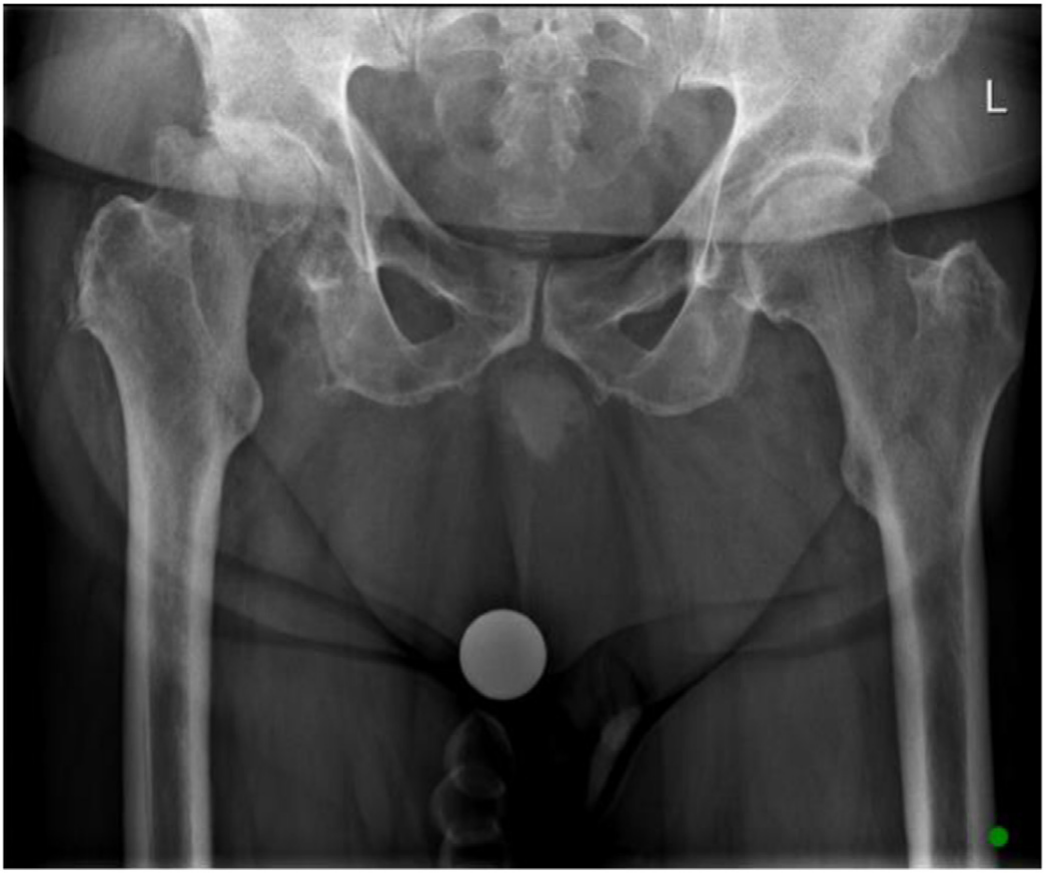
G1, pannus above the pubic symphysis.

**Figure 3:**
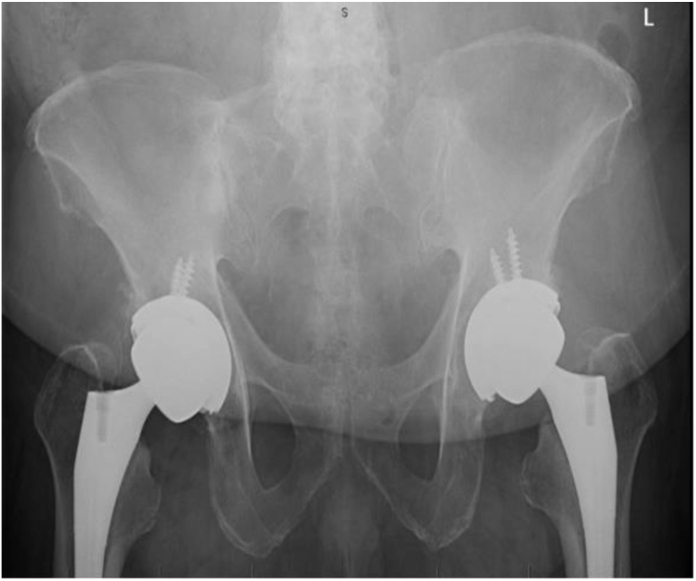
G2, pannus below the pubic symphysis, but above the ischial tuberosities.

**Figure 4:**
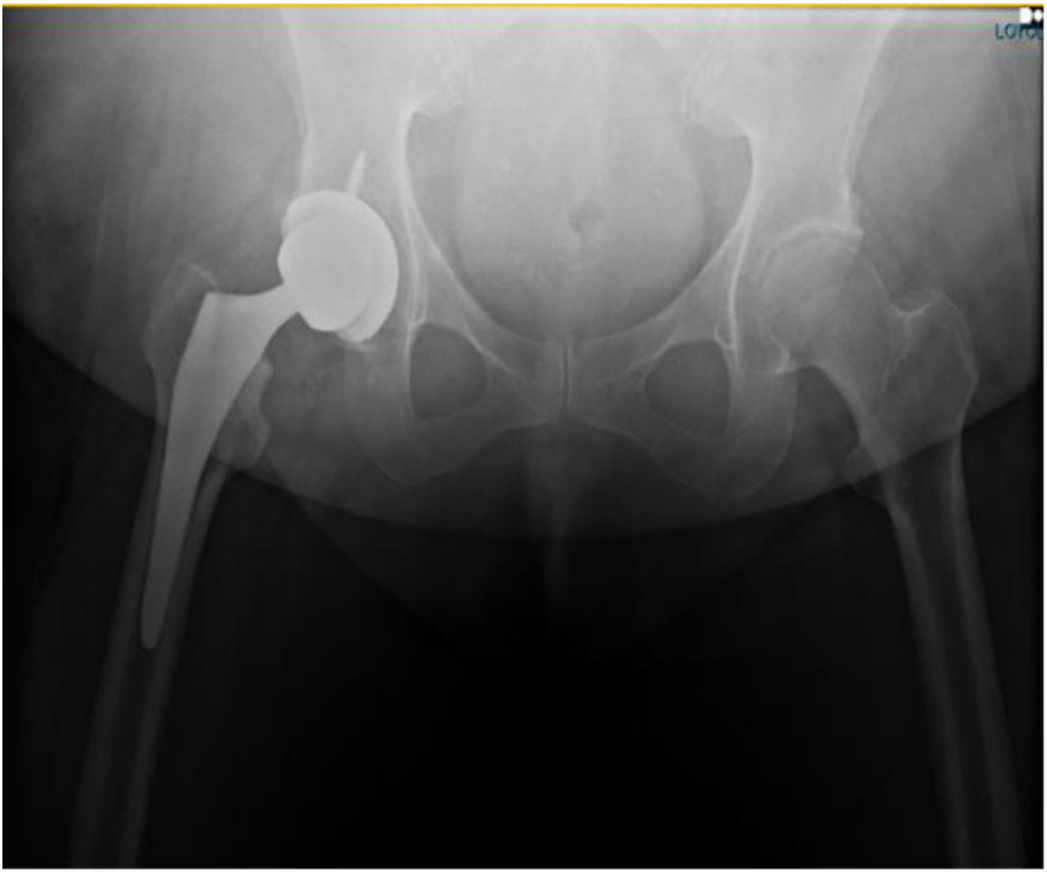
G3, pannus below the ischial tuberosities.

This was an institutional review board–approved, retrospective study of 1000 consecutive primary THA patients (478 anterior and 522 posterior approaches) performed by 4 surgeons at an academic tertiary referral center from 2017-2021. Two of the surgeons predominantly performed anterior approach and 2 of the surgeons predominantly performed posterior approach. In few instances, the anterior surgeons just used posterior approaches for conversions, revisions, or surgeries involving complex acetabular deformity. Present study excluded conversions revisions, and only included primary THA. All patients with the anterior approach had a standard longitudinal anterior skin incision. There were no formal criteria that determined what approach would be used for a patient. This study reviewed postoperative complications such as postoperative infections, reoperation within 90 ds, and antibiotic usage. Return to operating room (OR) within 90 ds only includes indications for infection and wound complications; other causes of reoperation such as instability, pain, and/or fractures were excluded from this category. Postoperative infections were noted as any delayed wound healing or “wound healing problem”. This was defined as anyone who had anything outside of the normal protocol for removing dressing and stitches/staples at their first postoperative visit, which included any follow-ups beyond the standard follow-up schedule for the wound, wound treatments, and/or antibiotic use. It should be noted that prophylactic antibiotics were not used in any of the patients. Abdominal pannus were radiographically measured by 1 orthopaedic resident on an anteroposterior pelvis image and placed into 1 of 4 categories based on its vertical size (no pannus, G0; Fig. 1), above symphysis (G1; Fig. 2), below symphysis (G2; Fig. 3), or below ischial tuberosities (G3; Fig. 4). Data retrieved from electronic medical records included patient demographics (sex, age, race, and BMI), Charlson comorbidity indices, and radiographic measurements using Inteleviewer or picture archiving and communication system.

Chi-squared tests for univariate and logistic regression models with forced entry model building were used for statistical analysis, controlling for age, race, gender, BMI, Charlson comorbidity index, and smoking.

## Results

Demographics of all included patients were 581 women and 419 men, mean age of 64.1 ys (standard deviation [SD] 10.4), mean BMI of 31.4 (SD 6.6), and mean Charlson comorbidity index of 4.5 (SD 3.9).

Overall, 611 patients had a pannus and 389 did not. Of the patients with anterior approach, 213 patients had no pannus, 138 had a grade 1, 67 had a grade 2, and 60 had a grade 3 pannus. Of the patients with posterior approach, 176 had no pannus, 150 had a grade 1, 86 had a grade 2, and 110 had a grade 3 pannus.

### Pannus vs no pannus

For patients without a pannus (G0), 4 of 213 patients (1.9%) had delayed wound healing in the anterior approach group vs 7 of 176 patients (3.9%) in the posterior approach group (*P* = .21). For patients with a noted pannus, 32 of 265 patients (12.1%) with anterior approach demonstrated delayed wound healing vs 37 of 346 patients (10.7%) with posterior approach (*P* = .58) (Table 1).

**Table 1:** Univariate analysis of wound complications (WCs) based on presence of pannus and approach (anterior vs posterior).

Pannus	Anterior (% with WC)	Posterior (% with WC)	*P* value
No	1.9	3.9	.21
Yes	12.1	10.7	.58

WC, wound complication.

For patients without a pannus (G0), 2 of 213 patients (0.9%) returned to the OR after anterior approach vs 2 of 176 patients (1.1%) after posterior approach (*P* = .28). For patients with a pannus, 5 of 265 patients (1.1%) returned to the OR after anterior approach vs 13 of 346 patients (3.8%) after posterior approach (*P* = .06) (Table 2).

**Table 2:** Univariate analysis of reoperations based on presence of pannus and approach (anterior vs posterior).

Pannus grade	Anterior (% with reoperations)	Posterior (% with reoperations)	*P* value
No	0.9	1.1	.08
Yes	1.1	3.8	.06

In the no pannus group, 4 of 213 patients (1.9%) required antibiotics after anterior approach vs 5 of 176 patients (2.8%) after posterior approach (*P* = .28). In the pannus group, 7 of 265 patients (2.6%) required antibiotics after anterior approach vs 21 of 346 patients (6.1%) after posterior approach (*P* < .05) (Table 3).

**Table 3:** Univariate analysis of antibiotic usage based on presence of pannus and approach (anterior vs posterior).

Pannus grade	Anterior (% with antibiotics)	Posterior (% with antibiotics)	*P* value
No	1.9	2.8	.28
Yes	2.6	6.1	.045^a^

a Indicates statistically significant difference (*P* < .05).

When controlling for BMI, age, race, gender, Charlson comorbidity index, and smoking in the logistic regression models, there was no statistically significant difference in the odds of wound complications (Table A1) or return to the OR (Table A2) between the approaches and presence of pannus. Specifically, there were 60 patients with noted rheumatoid arthritis (6%). Of these patients, 20 were without pannus, 21 with pannus grade 1, 13 with pannus grade 2, and 6 with pannus grade 3. Only patients with grade 2 and grade 3 pannus demonstrated delayed wound healing (3 patients each), return to OR (1 patient each), and antibiotic use after surgery (1 patient each).

Post-hoc power analysis was performed to determine validity of results. With power threshold (1-β) set at 0.8 and significance (α) of 0.05, the power of this study was calculated to be 0.23 for delayed wound healing between approaches in the no pannus group, and 0.08 between approaches in the pannus groups; 0.04 for return to OR between approaches in the no pannus group, and 0.55 between approaches in the pannus groups; and 0.09 for antibiotic use between approaches in the no pannus group, and 0.54 between approaches in the pannus groups.

### Interpannus group comparisons

In the G1 pannus group, there were 10 patients (7.2%) who demonstrated wound healing problems in the anterior approach group vs 10 patients (6.7%) in the posterior approach group (*P* = .08). In the G2 pannus group, delayed wound healing was found in 12 of 67 patients (17.9%) with anterior approach vs 10 of 86 patients (11.6%) with posterior approach (*P* = .27). The G3 pannus group showed this in 10 of 60 patients (16.7%) with anterior approach vs 17 of 110 patients (15.5%) with posterior approach (*P* = .84) (Table 4).

**Table 4:** Univariate analysis of wound complications (WCs) based on pannus grade and approach.

Pannus grade	Anterior (% with WC)	Posterior (% with WC)	*P* value
G0	1.9	3.9	.21
G1	7.2	6.7	.08
G2	17.9	11.6	.27
G3	16.7	15.5	.84

WC, wound complication.

Patients with a G1 pannus returned to the OR in 2 of 136 cases (1.4%) with anterior approach vs 3 of 147 (2%) in posterior approach (*P* = .72). Those with a G2 pannus returned in 2 of 65 cases (3.0%) vs 5 of 81 cases (5.8%) (*P* = .41). Finally, patients with a G3 pannus returned to the OR 1 of 59 cases (1.7%) with the anterior approach vs 5 of 105 cases (4.5%) with posterior approach (*P* = .33) (Table 5).

**Table 5:** Univariate analysis of reoperations based on pannus grade and approach.

Pannus grade	Anterior (% with reoperations)	Posterior (% with reoperations)	*P* value
G0	0.9	1.1	.08
G1	1.4	2.0	.72
G2	3.0	5.8	.41
G3	1.7	4.5	.33

Additionally, when controlling for BMI, age, race, gender, Charlson comorbidity index, and smoking in the logistic regression models, there was no statistically significant difference in the odds of wound complications (Table A3) or return to the OR (Table A4) between the approaches at each pannus size.

## Discussion

A large, overhanging pannus has once been proposed as a contraindication to direct anterior approach for THA given its elevated risks of complications and infections [2,3]. It has been proposed that the approach to THA may not actually be increasing the rates of complications but rather that patients with pannus or elevated BMIs are just more likely to suffer perioperative and/or postoperative complications [4,9]. Prior literature has noted mixed results when looking into complications and BMI. In 2013, it was suggested that a BMI cutoff of > 40 would benefit patients whose perioperative complications would outweigh the benefits of joint replacement [10]. Future studies then found that arthroplasty in patients with > 40 BMI did not confer with significantly greater complication risk, and that implementation of BMI cutoffs for surgery hindered patients from achieving quality improvements via surgical intervention [11,12]. Therefore, BMI alone should not dictate the course of surgical vs conservative management; rather, a combination of concomitant comorbidities and shared decision-making should aid in the best course of action [12]. Nonetheless, this study stratified patients by pannus rather than BMI, but controlled for BMI. Other studies had looked at BMI but the purpose of this was to look at the patients’ pannus which, while influenced by BMI, isn't always directly correlated [12]. Furthermore, by including all BMI levels, this allowed us to evaluate differing levels of pannus size.

The results of this study demonstrated no significant difference in infection rates between approaches, across the pannus and no pannus groups. Patients without a pannus showed no significant differences in the rate of antibiotic use after either approach; however, patients with a pannus required antibiotics at a significantly higher rate when using the posterior approach. Although it should be noted that requiring antibiotics is likely a surgeon preference, there should be no bias given the heterogenous group of patients and surgeons in this study. The size of a pannus did not confer a statistically significant correlation with increased complications using one approach over the other. However, of the 1000 patients included in this study, there was a higher prevalence of delayed wound healing and reoperation in patients with a pannus regardless of the approach. These results support prior notions of the theory that postulates people with pannus may have immune dysregulation that may make them more susceptible to infections rather than the infection risk being due to the approach [1,8,9].

Limitations largely rest on retrospective nature of this study, as well as the variability in operative technique and management of the 4 different surgeons included in this study. Of the 4 surgeons, 2 exclusively performed posterior approach and 2 predominantly performed the anterior approach. However, given this is a retrospective study, there is still risk of selection bias. As a retrospective study, there was also no uniform method of closure, and all surgeons used different closure methods. Additionally, there were no data on the usage of incisional wound vacuum assisted closures as this info is very specifically documented; nonetheless, it is very rarely used in the primary setting at this institution. Furthermore, most of the images were supine, but some were standing which may have influenced the pannus measurements. All patients included in this study were operated at a single center; results may be skewed based on hospital or geographical factors. Although the univariate analysis demonstrated higher wound complications for the anterior approach group in the interpannus comparisons, this study was underpowered to show true associations; it should be noted that the multivariate regression model also noted no significant wound complication rate differences between approaches. Finally, the significantly increased rate of antibiotic use in the pannus group after posterior approach may be due to selection bias per surgeon, as some may have a lower threshold as to when to treat patients with antibiotics, possibly falsely creating a significant bias in this regard.

## Conclusions

Presence and size of abdominal pannus should not dictate surgical approach. It itself does not change outcomes regarding postoperative complications, and infections in either anterior or posterior approaches. While there may be many factors involved in deciding surgical approach per individual patient, surgeon, or practice, both anterior and posterior approaches are safe options regardless of pannus size.

## Funding

This research did not receive any specific grant from funding agencies in the public, commercial, or not-for-profit sectors.

## Conflicts of interest

Nicholas Brown received royalties from Corin and Link; is a paid consultant for Depuy, Corin, Link, and Smith & Nephew; and is a board member in the AAOS. All other authors declare no potential conflicts of interest.

For full disclosure statements refer to https://doi.org/10.1016/j.artd.2025.101675.

## CRediT authorship contribution statement

**Samantha Bialek:** Visualization, Validation, Methodology, Investigation, Data curation, Conceptualization. **William Oetojo:** Writing – review & editing, Writing – original draft, Validation, Formal analysis. **Robert Burnham:** Validation, Supervision, Project administration, Investigation, Data curation, Conceptualization. **Nicholas Brown:** Writing – review & editing, Writing – original draft, Validation, Supervision, Project administration, Formal analysis, Conceptualization.

## Figures and Tables

**Table A1:** Logistic regression analysis of odds ratio (OR) of wound complications (WCs) based on approach and presence of pannus.

Pannus	Approach	OR	CI	*P* value
No	Anterior	0.47	0.15-1.49	.20
Yes	Anterior	1.60	0.94-2.75	.09
No	Posterior	0.92	0.36-2.73	.87
Yes	Posterior	Reference		.09

Regression analysis controlled for BMI, age, race, gender, Charlson comorbidity index, and smoking.

CI, confidence interval; OR, odds ratio.

**Table A2:** Logistic regression analysis of odds ratio (OR) of reoperations based on approach and presence of pannus.

Pannus grade	Approach	OR	CI	*P* value
No	Anterior	0.58	0.10-3.26	.54
Yes	Anterior	0.63	0.21-1.85	.40
No	Posterior	0.69	0.13-3.81	.67
Yes	Posterior	Reference		.83

Regression analysis controlled for BMI, age, race, gender, Charlson comorbidity index, and smoking.

CI, confidence interval; OR, odds ratio.

**Table A3:** Logistic regression analysis of odds ratio (OR) of wound complications (WCs) based on approach and interpannus comparisons.

Pannus grade	Approach	OR	CI	*P* value
G0	Anterior	0.39	0.11-1.42	.15
G1	Anterior	1.07	0.42-2.78	.88
G2	Anterior	2.01	0.83-4.85	.12
G3	Anterior	1.30	0.53-3.17	.56
G0	Posterior	0.77	0.25-2.33	.64
G1	Posterior	0.74	0.30-1.80	.50
G2	Posterior	0.92	0.38-2.24	.86
G3	Posterior	Reference		.24

Regression analysis controlled for BMI, age, race, gender, Charlson comorbidity index, and smoking.

CI, confidence interval; OR, odds ratio.

**Table A4:** Logistic regression analysis of odds ratio (OR) of reoperations based on approach and interpannus comparisons.

Pannus grade	Approach	OR	CI	*P* value
G0	Anterior	0.62	0.08-4.82	.65
G1	Anterior	0.67	0.10-4.30	.67
G2	Anterior	1.04	0.18-6.03	.97
G3	Anterior	0.42	0.05-3.79	.44
G0	Posterior	0.75	0.10-5.56	.78
G1	Posterior	0.75	0.15-3.61	.72
G2	Posterior	1.77	0.46-6.82	.41
G3	Posterior	Reference		.89

Regression analysis controlled for BMI, age, race, gender, Charlson comorbidity index, and smoking.

CI, confidence interval; OR, odds ratio.

## Appendix A Supplementary data

Conflict of Interest Statement for Brown

Conflict of Interest Statement for Bialek

Conflict of Interest Statement for Burnham

Conflict of Interest Statement for Oetojo
