# Supplementary material for: Abdominal Pannus Should Not Dictate Surgical Approach in Primary Total Hip Arthroplasty
Source: Arthroplast Today. 2025 Mar 26;33:101675. doi: 10.1016/j.artd.2025.101675 (PMC11986245; doi:10.1016/j.artd.2025.101675)
Supplement: Conflict of Interest Statement for Burnham [file mmc3.pdf]

INDIVIDUAL CONFLICT OF INTEREST STATEMENT

American Association of Hip and Knee Surgeons

(Adopted from the American Academy of Orthopaedic Surgeons disclosure statement)

The following form **<u>must be filled out completely and submitted by each author (example, 6 authors, 6 forms).</u>**
<u>All items require a response. If there is no relevant disclosure for a given item, enter "*None.*"</u>

Manuscript Title: Abdominal Pannus Should Not Dictate Surgical Approach in Primary Total Hip Arthroplasty

1. Royalties from a company or supplier (The following conflicts were disclosed)

None

2. Speakers bureau/paid presentations for a company or supplier (The following conflicts were disclosed)

None

3A. Paid employee for a company or supplier (The following conflicts were disclosed)

None

3B. Paid consultant for a company or supplier (The following conflicts were disclosed)

None

3C. Unpaid consultants for a company or supplier (The following conflicts were disclosed)

None

4. Stock or stock options in a company or supplier (The following conflicts were disclosed)

None

5. Research support from a company or supplier as a Principal Investigator (The following conflicts were disclosed)

None

6. Other financial or material support from a company or supplier (The following conflicts were disclosed)

None

7. Royalties, financial or material support from publishers (The following conflicts were disclosed)

None

8. Medical/Orthopaedic publications editorial/governing board (The following conflicts were disclosed)

None

9. Board member/committee appointments for a society (The following conflicts were disclosed)

None

<u>Each author must sign AND print or type his/her name, date and submit a separate form</u>

In addition, one BLINDED Conflict of Interest form (no author names used) should be submitted per manuscript with all author disclosures.

Robert Burnham	Robert Burnham Jr.	3/5/2024
Author Name (Print or Type)	Author Signature	Date
